# Intrarenal Transplantation of Hypoxic Preconditioned Mesenchymal Stem Cells Improves Glomerulonephritis through Anti-Oxidation, Anti-ER Stress, Anti-Inflammation, Anti-Apoptosis, and Anti-Autophagy

**DOI:** 10.3390/antiox9010002

**Published:** 2019-12-18

**Authors:** Hao-Hsiang Chang, Shih-Ping Hsu, Chiang-Ting Chien

**Affiliations:** 1 School of Life Science, National Taiwan Normal University, Taipei 116, Taiwan; allanchanghs@gmail.com; 2 Department of Family Medicine, National Taiwan University Hospital and College of Medicine, Taipei 100, Taiwan; 3 Department of Internal Medicine, Far Eastern Memorial Hospital, New Taipei City 220, Taiwan

**Keywords:** apoptosis, autophagy, hypoxic preconditioning, mesenchymal stem cell, Nrf2, inflammation

## Abstract

To confer further therapeutic potential and prevent some adverse effects by the mesenchymal stem cells (MSCs) transplantation, we explored the effects of locally intrarenal arterial administration of hypoxic preconditioned MSCs in the anti-Thy1.1 induced rat glomerulonephritis. Proteinuria, histochemical staining, and western blotting were used to explore the therapeutic effects and mechanisms. Locally intrarenal arterial MSCs transplantation successfully implanted the fluorescent or CD44 labeled MSCs in the nephritic glomeruli, ameliorated proteinuria, and glomerulosclerosis in nephritic rats. Hypoxic preconditioning significantly upregulated hypoxic inducible factor-1α/VEGF (HIF-1α/VEGF) in the MSCs and was more efficient than normoxic MSCs in reducing the degree of urinary protein, glomerulosclerosis, fibrosis, macrophage/monocyte infiltration, GRP78 mediated endoplasmic reticulum stress, Beclin-1/LC3-II mediated autophagy, and Bax/Bcl-2/caspase 3 mediated apoptosis. Hypoxic MSCs could further promote intranuclear nuclear factor (erythroid-derived 2, Nrf2) and reduce nuclear factor kappa B expression in nephritic kidneys. As compared to normoxic MSCs, hypoxic MSCs transplantation significantly upregulated the renal expression of anti-oxidative response elements/enzymes including glutamate-cysteine ligase catalytic subunit, glutamate-cysteine ligase modifier subunit, glutathione peroxidase, catalase, Mn, and Cu/Zn superoxide dismutase. In summary, intrarenal hypoxic preconditioning MSCs transplantation was more effective to activate hypoxic inducible factor-1α/VEGF/Nrf2 (HIF-1α/VEGF/Nrf2) signaling, preserve anti-oxidant proteins and anti-oxidative responsive element proteins, and subsequently reduce glomerular apoptosis, autophagy, and inflammation.

## 1. Introduction

Glomerulonephritis (GN) is a constellation of heterogenous renal diseases featured as a shared pathophysiology of immune mediated glomerular inflammation [1,2]. Some GN patients may benefit from specific immunosuppressive therapies and many others who are irresponsive to this type of management eventually develop end stage renal disease (ESRD) [3,4,5,6]. Glomerulonephritis is one of the leading causes of ESRD and remains a medical challenge [7]. Implementation of interventions to cure or prevent GN related deterioration is very important from both public health and economic aspects. Stem cells have the potential of self-renewal and the ability of immune modulation and as such, provide a potential therapeutic option to the unmet need of GN sufferers [8].

Mesenchymal stem cells (MSCs) have been shown to improve renal functions in animal GN models [8], and in some refractory human lupus nephritis studies [9,10,11]. In these studies, MSCs cross-talked with target organs by secreting growth factors, cytokines, and prostaglandins, which regulate anti-inflammation, anti-apoptosis, and anti-fibrosis effects and enhance cell proliferation, survival, and angiogenesis to repair injured tissue. These paracrine effects play a major role in MSC’s therapeutic effect to the damaged kidney [2,8,12]. Additionally, increased oxidative stress contributes to the pathogenesis of mesangial proliferative GN and leads to renal dysfunction [13]. MSCs have been shown to possess anti-oxidative effects which help in the treatment of GN [14]. Strategies to enhance their anti-oxidative ability could promote the therapeutic efficacy of MSCs [15]. However, compared with the anti-inflammatory and immune-modulatory effects, the mechanisms underlying the anti-oxidative effects are relatively unknown. Hypoxic preconditioning is a promising strategy to improve the efficacy and stemness of MSC therapy, through preventing senescence, increasing differentiation efficiency [16], and enhancing MSC homing [17]. There is surprisingly little knowledge about how hypoxic preconditioning affects the anti-oxidative therapeutic effects of MSCs. The present study aimed to investigate the anti-oxidative stress mechanisms involved in the use of locally intrarenal transplantation, for reduction of possible adverse effect, of normoxic MSCs and hypoxic-preconditioned mesenchymal stem cells (HMSCs) in the anti-thy1.1 GN rat model.

## 2. Materials and Methods

### 2.1. Animals

Female Wistar rats weighing 220–250 g were purchased from BioLASCO Taiwan Co. Ltd. (Taipei, Taiwan) and housed in the Experimental Animal Center, National Taiwan University. All the surgical and experimental procedures were approved by the ethics committee “Institutional Animal Care and Use Committee of the National Taiwan University College of Science” (identification code of approval: 20100244 and date of approval: 02/11/2011) and were in accordance with the guidelines of the National Science Council of Republic of China (NSC 1997). To monitor fecal and urinary excretion, the rats were placed into the metabolic cage. The feces and urine samples were collected and recorded every 12 h before surgical experiments. During the experiment, the rats were given free access to food and water.

### 2.2. Cell Preparations (MSCS Isolation, Characterization, and Culture)

Femora from Wistar rats (BioLASCO Taiwan Co Ltd., Taipei, Taiwan), 8 to 10 weeks of age, were removed, and soft tissues were detached aseptically. Bone marrow was extruded by inserting a 23-gauge needle into the shaft of the bone and was flushed out with basal medium (α-minimal essential medium [α-MEM], Gibco-BRL, Gaithersburg, MD, USA). Isolation of MSCs was performed according to similar procedures as described previously [18]. Briefly, mononuclear cells were isolated from the bone marrow aspirates by a density gradient centrifugation method were suspended in complete culture medium (α-MEM supplemented with 16.6% fetal bovine serum, 100 U/mL penicillin, 100 μg/mL streptomycin, and 2 mM L-glutamine) and seeded in plastic dishes. After 24 h of the initial culture, nonadherent cells were removed by a change of medium and irrigation of the culture. The culture typically reaches 65% to 70% confluency within 6 to 8 days and reached subconfluency at 9 days, when the cells (passage 0) were harvested for further subculturing. Starting from passage 1, the cells were seeded at 100 cells/cm^2^ and were grown in complete culture medium with a medium change twice per week. For hypoxic MSC cultures, cells were cultured in a gas mixture composed of 94% N_2_, 5% CO_2_, and 1% O_2_ [19], whereas in normoxic MSC cultures, cells were cultured in 95% air and 5% CO_2_. For maintenance of the hypoxic gas mixture, an incubator with two air sensors, one for CO_2_ and the other for O_2_, was used. O_2_ concentration was achieved and maintained using delivery of N_2_ gas from a tank containing pure N_2_. If the O_2_ concentration rose above the desired level, N_2_ gas was automatically injected into the system to displace the excess O_2_.

### 2.3. HIF-1α Determination and Growth Factors Array Assay

The hypoxic inducible factor-1α (HIF-1α) concentration and multiple growth factors assay from cultured condition medium and MSCs or HMSCs were determined with HIF-1α ELISA kit (MBS2702491, MyBioSource, San Diego, CA, USA) and RayBio^®^ rat growth factor array (AAR-GF-1-2, RayBiotech, Peachtree Corners, GA, USA) according to the manufacturer’s instructions.

### 2.4. Experimental Model and Design

**Figure 1:**
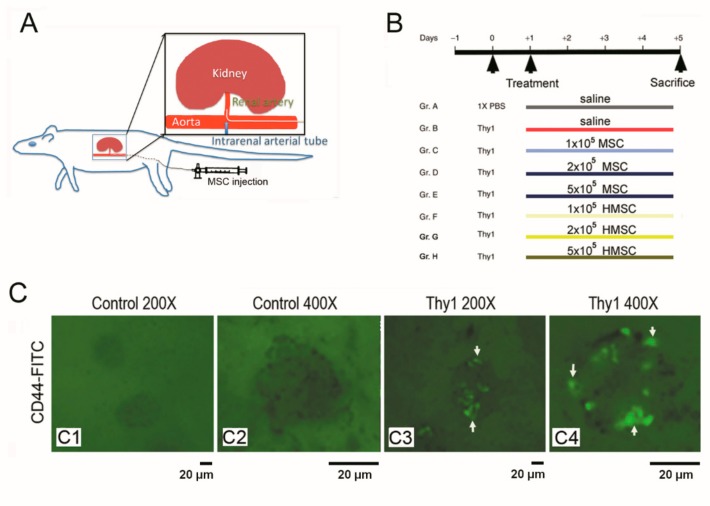
The technique for locally intra-renal arterial administration of mesenchymal stem cells (MSCs) was displayed in (**A**). The experimental grouping and design are displayed in the eight groups (**B**). High levels of green fluorescence were visualized under UV light in the glomeruli of nephritic kidney sections (**C3–4**) but not in the normal kidney sections (**C1–2**). C1, C3 magnification 200×; C2, C4 magnification 400×.

Anti-thy1.1 GN was induced by injection of 0.2 mL of phosphate-buffered saline containing 250 μg anti-thy1.1 monoclonal antibody (Cedarlane, Burlington, ON, Canada) into rats via a jugular vein under sodium pentobarbital anesthesia (50 mg/kg, i.p.) at day 0, and 0.2 mL of saline injection into the jugular vein as a control group. This method for induction of acute GN had been reported previously [20]. Under avertin anesthesia (400 mg/kg, Acros Organics, Morris Plains, NJ, USA), one PE10 tubing was introduced into the left renal artery from the left femoral artery via the aorta for direct MSCs or HMSCs delivery (Figure 1A). Varied numbers of MSCs or HMSCs including 1, 2, and 5 × 10^5^ cells were administered via this catheter in the therapeutic groups, and saline was administration in control groups. The grouping and experimental design are shown in Figure 1B.

### 2.5. Tracking of Intrarenal Arterial Injected MSCs in Rat Kidneys

To ascertain the MSC expression in the kidney, we infused MSCs containing a green fluorescent protein (GFP) into the left kidney and examined the GFP expression in rat kidneys one hour later. The sections were examined under UV light for the detection of fluorescence around the glomeruli, arterial lining cells and tubular cells. Immunochemical stains with primary antibodies against MSC CD44 (MCA643GA, Serotec, Kidlington, UK) were also performed for identification of MSCs in kidneys.

### 2.6. Measurements of Proteinuria and Hydroxyproline Degree

Twenty four hours urine samples were collected (on day 5 after anti-Thy1.1 infusion) from all experimental rats with free access to water. Urinary protein concentration was determined by a Bio-Rad protein assay (Bio-Rad Laboratories, München, Germany). Hydroxyproline content was measured with Hydroxyproline Assay Kit (STA-675, Cell Biolabs, Inc., San Diego, CA, USA).

### 2.7. Renal Morphology

For assessing the morphological change, renal histology was evaluated using H&E, periodic acid-Schiff (PAS) and Masson stained 5-µm paraffin sections based on at least 50 glomeruli per kidney section. PAS-stained sections were examined for glomerulosclerosis. One hundred glomeruli per section were randomly selected for assessing the degree of glomerular damage using a semi-quantitative scoring method: grade 0, normal glomeruli; grade 1, sclerotic area up to 25% (minimal sclerosis); grade 2, sclerotic area 25 to 50% (moderate sclerosis); grade 3, sclerotic area 50 to 75% (moderate-severe sclerosis); grade 4, sclerotic area 75 to 100% (severe sclerosis), as described previously [21].

### 2.8. Immunohistochemistry

Immunohistochemical staining was performed on formalin fixed, paraffin-embedded kidney sections with ED-1 (clone ED-1, Serotec, Oxford, UK), primary antibodies against MSC CD44 (MCA643GA, Serotec, Kidlington, UK), GRP78 (1:500; Santa Cruz Biotechnology, Dallas, TX, USA), LC3-II (1:1000; MBLI Corporation, Woburn, MA, USA), caspase 3 (Epitomics, Burlingame, CA, USA), terminal deoxynucleotidyl transferase-mediated digoxigenin-deoxyuridine nick-end labeling (TUNEL, BioVision, Milpitas, CA, USA), collagen IV (Abcam, Cambridge, UK) and 4-hydroxynonenal (4HNE, Bioss, Woburn, MA, USA). Briefly, paraffin sections were deparaffinized with xylene and rehydrated in an alcohol series and water. Kidney sections were subjected to antigen retrieval and were blocked with a peroxidase-blocking reagent. Sections were incubated with the primary antibody overnight at 4 °C. After washing, the kidney sections were incubated with Envision system-horseradish peroxidase-labeled polymer (Dako, Glostrup, Denmark) for 1 h at room temperature. The sections were visualized with 3,3′-diaminobenzidine tetrahydrochloride (Dako, Glostrup, Denmark) and counterstained with hematoxylin. Apoptotic cells in the kidney were identified by TUNEL staining. The TUNEL method for the in situ apoptotic assay was performed according to the method of Gavrieli et al. with minor modifications [22]. The number of positive ED1 and TUNEL stained cells was evaluated by counting stained cells per high power field (×400) in at least 20 randomly selected fields. The percentage of positive stained area in the GRP-78, LC3-II, caspase 3 and collagen VI assays was analyzed by Adobe Photoshop 7.0.1 imaging software (San Jose, CA, USA) analysis.

### 2.9. Western Blot and Nuclear Extraction

Western blot analysis was performed on isolated glomeruli to detect the levels of renal anti-oxidant responsive element proteins, and nuclear extractions were done to detect nuclear factor (erythroid-derived 2)-like 2 (Nrf2), nuclear factor kappa B (NF-kB) expressions. Briefly, tissues were grinded to powder in liquid nitrogen. Then the tissue powder was lysed in RIPA Buffer (Bio Basic, Amherst, NY, USA) supplemented with a protease inhibitor (Roche, Basel, Switzerland) for 10 min at 4 °C. The concentration of protein was measured by a BCA protein assay kit (Thermo Scientific, Waltham, MA, USA). A protein sample (80 μg) was mixed with 1× sample buffer and was boiled for 3 min. Protein samples were resolved in 10% SDS-polyacrylamide gel electrophoresis (SDS-PAGE) and transferred to PVDF membrane (Millipore, Billerica, MA, USA). The blot was blocked with Hyblock (Hycell, Taipei, Taiwan) for 1 min, and incubated with primary antibodies overnight at 4 °C. Detection of signals was performed by Western Lightning plus-ECL (PerkinElmer, Waltham, MA, USA). Nuclear extracts were obtained using the NE-PER nuclear and cytoplasmic extraction reagents (Thermo Scientific, Waltham, MA, USA) according to the manufacturer’s instructions.

Primary antibodies included Mn-superoxide dismutase (MnSOD, 1:1000; Enzo Life Sciences, Farmingdale, NY, USA), Cu/Zn-SOD (1:500; Millipore, Billerica, MA, USA), catalase (Assay Designs, Ann Arbor, MI, USA), heme oxygenase 1(BioVision, Milpitas, CA, USA), Nrf2 (Cayman, Ann Arbor, MI, USA), NFkB (Santa Cruz, Dallas, TX, USA), glutamate-cysteine ligase catalytic subunit (GCLC, Abcam, Cambridge, UK), glutamate-cysteine ligase modifier subunit (GCLM, Abcam, Cambridge, UK), glutathione peroxidase 1(GPX1, Abcam, Cambridge, UK), β-actin (1:5000; Sigma-Aldrich, St. Louis, MO, USA), γ-tubulin (Abcam, Cambridge, UK) and Lamin A/C (Abcam, Cambridge, UK) as a control for nuclear extraction. Secondary antibodies included HRP-conjugated goat anti-mouse IgG, HRP-conjugated rabbit anti-goat IgG, and HRP-conjugated goat anti-rabbit IgG (all at 1:10,000; all from Southern Biotech Laboratories, Birmingham, AL, USA).

### 2.10. Statistical Analysis

We used the soft Scion Image β3b Scion Corporation, Frederick, MD software to quantify western blot density. We used a one-way ANOVA and Bonferroni SPSS/Windows (SPSS Inc., Chicago, IL, USA) to analyze experimental data (the difference from the experimental was assessed by a one way ANOVA). We used GraphPad PRISM^®^ 3.0 (GraphPad Software Inc., San Diego, CA, USA) and Sigma Plot 10.0 (San Jose, CA, USA) for figure preparation.

## 3. Results

### 3.1. Recruitment of MSCs and HMSCs into Nephritic Not Normal Kidneys

**Figure 2:**
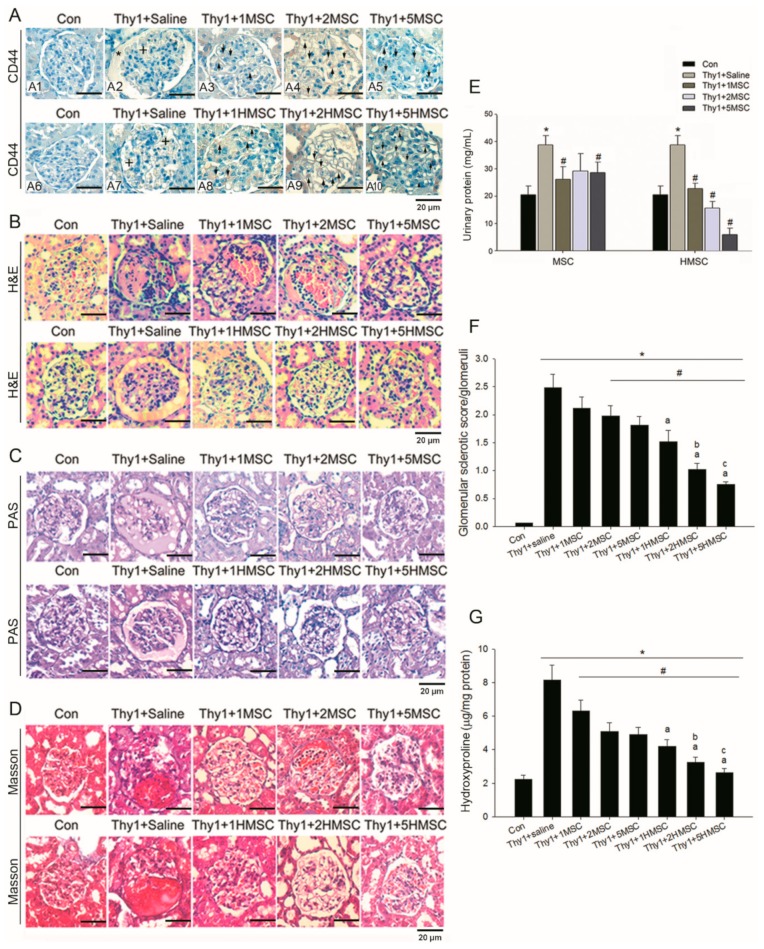
Representative pictures of immunohistochemical stained sections for CD44, a marker for MSC, in ten groups of rats show CD44 positive stains in the glomeruli indicated by arrows in MSCs or HMSCs treated kidneys compared to control kidney (**A1–10**). Anti-Thy1.1 evoked mesangial lysis (indicated by +) and the mesangial matrix accumulation (indicated with *) in the nephritic rats. Intrarenal MSCs transplantation ameliorated anti-Thy1.1-induced nephritis in the rat model. Normoxic (MSC) and hypoxic MSCs (HMSC) markedly reduced the inflammatory cell infiltration in the glomeruli in H&E stains (**B**). The severity of glomerulosclerosis was explored in both normoxic MSC and HMSC treated rats in PAS stains (**C**) and in Masson stains (**D**). Thy1-induced nephritis significantly elevated urinary protein level and the elevated urinary protein level was significantly reduced by intrarenal MSC or HMSC treatment (**E**). Glomerular sclerotic index calculated by the PAS sections among the experimental groups are presented in (**F**). Thy1-induced nephritis significantly increased glomerular sclerosis in all nephritis treated groups as compared to Con group. MSC at cell number (2–5) × 10^5^ level and HMSC at cell number (1–5) × 10^5^ level significantly reduced sclerosis degree. Glomerular fibrosis determined by the hydroxyproline contents among all groups of animals are presented in (**G**). Thy1-induced nephritis significantly increased renal hydroxyproline content in all nephritis treated groups as compared to Con group. MSC and HMSC significantly reduced renal fibrotic degree. Each graph is amplified at 400×. The scale bar (20 μm) is indicated in each graph. * *p* < 0.05 vs. Con group. # *p* < 0.05 vs. Thy1 group. a *p* < 0.05 vs. Thy1 + 1MSC group. b *p* < 0.05 vs. Thy1 + 2MSC group. c *p* < 0.05 vs. Thy1 + 5MSC group.

Detection of fluorescence in the kidneys after intrarenal arterial administration of fluorescent MSCs is illustrated in Figure 1C. High levels of GFP were visualized under UV light and the fluorescence was found in the nephritic kidneys (Figure 1C3,4) but not in the normal kidney (Figure 1C1,2). Fluorescence cells were found at glomeruli in the MSC treated groups. CD44 positive cells were found in glomeruli of MSC or HMSC treated kidneys, but not in the control kidney (Figure 2A). These results confirm the recruitment of MSCs and HMSCs in injured kidneys. There was no significant difference in trafficking CD44 positive cell numbers between the MSC and HMSC groups.

### 3.2. MSC or HMSC Ameliorates Nephritic Severity in the Rat GN Model

The success of glomerulonephritis induction in our model was confirmed by the elevated urine protein concentrations and typical characteristics in the histopathologic examination, including mesangial cell proliferation, mesangialysis, and sclerosis appearance 5 days after injury. In H&E stains of both MSC and HMSC treated kidneys, the severity of glomerulosclerosis was attenuated and decreased numbers of inflammatory cells in glomeruli were found (Figure 2B). The glomerulosclerosis index by PAS stains sections (Figure 2C) are 0.01 for the control, 0.5 for anti-Thy1 with placebo, 0.19 for 1 × 10^5^ MSC treated group, 0.16 for 2 × 10^5^ MSC treated group, 0.03 for 5 × 10^5^ MSC treated group, 0.03 for 1 × 10^5^ HMSC treated group, and 0 for both 2 and 5 × 10^5^ MSC treated groups (Figure 2F). Both normoxic and hypoxic MSC reduced the severity of proteinuria in GN rats. The level of proteinuria reduction did not differ among 1, 2, 5 × 10^5^ MSC infusion, but there exists a trend in HMSCs with a higher cell number having a lower proteinuria (Figure 2E). The severity of glomeruli fibrosis was also ameliorated by MSC and HMSC administration, which is shown in the Masson stains (Figure 2D) and in the hydroxyproline content (Figure 2H).

### 3.3. Hypoxic Preconditioning Upregulated HIF-1α and VEGF Expression

**Figure 3:**
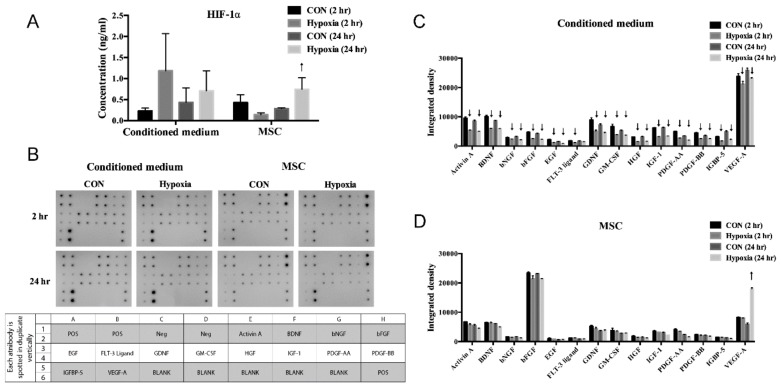
The concentration of HIF-1α (**A**) and multiple growth factors array (**B–D**) from conditioned medium and MSC were determined. The upregulation and downregulation were denoted with the arrows.

Hypoxic preconditioning significantly upregulated HIF-1α concentration (Figure 3A) and VEGF (Figure 3D) expression in the MSCs but downregulated several growth factors with growth factors array assay (Figure 3B) in the conditioned medium (Figure 3C).

### 3.4. MSCs or HMSCs Reduce ED1, ER Stress, Autophagy, and Apoptosis with Western Blot

**Figure 4:**
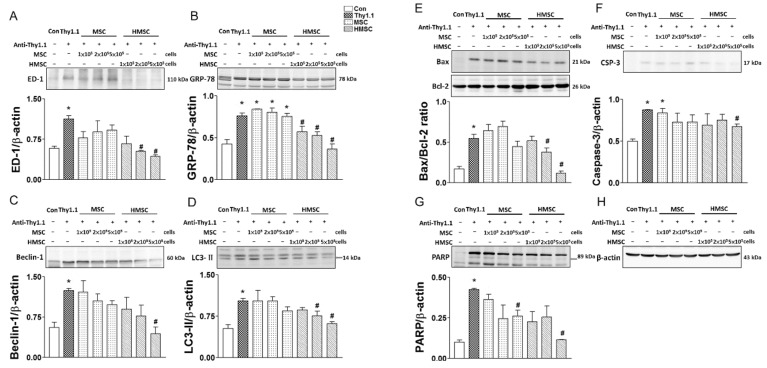
The oxidative stress index of renal ED-1 (**A**), GRP78 (**B**), Beclin-1 (**C**), LC3-II (**D**), Bax/Bcl-2 ratio (**E**), Caspase-3 (**F**), PARP (**G**) and β-actin (**H**) is determined with western blot among eight groups of rats. The quantitative data showed that anti-Thy1.1 significantly enhanced ED-1, GRP78, Beclin-1, LC3-II, Bax/Bcl-2 ratio, Caspase 3, and PARP in the damaged kidneys. MSCs with three amounts did not significantly reduce these oxidative parameters in the anti-thy1.1 treated kidneys. HMSCs treated anti-thy1.1 kidneys with the number of 5 × 10^5^ significantly reduced these parameters as compared to anti-thy1.1 treated group. * *p* < 0.05 vs. Con group. # *p* < 0.05 vs. Thy1.1 group.

The oxidative stress index of renal ED-1 (Figure 4A), GRP78 (Figure 4B), Beclin-1 (Figure 4C), LC3-II (Figure 4D), Bax/Bcl-2 ratio (Figure 4E), Caspase-3 (Figure 4F), PARP (Figure 4G) was significantly elevated in anti-Thy1.1 treated glomeruli. MSCs with three dosages did not significantly reduce these oxidative parameters in the anti-thy1.1 treated kidneys. However, HMSCs treated anti-thy1.1 kidneys with the number of 5 × 10^5^ significantly reduced these parameters as compared to anti-thy1.1 treated group.

### 3.5. MSCs or HMSCs Reduce ED-1 Infiltration, ER Stress, Autophagy, and Apoptosis by IHC

**Figure 5:**
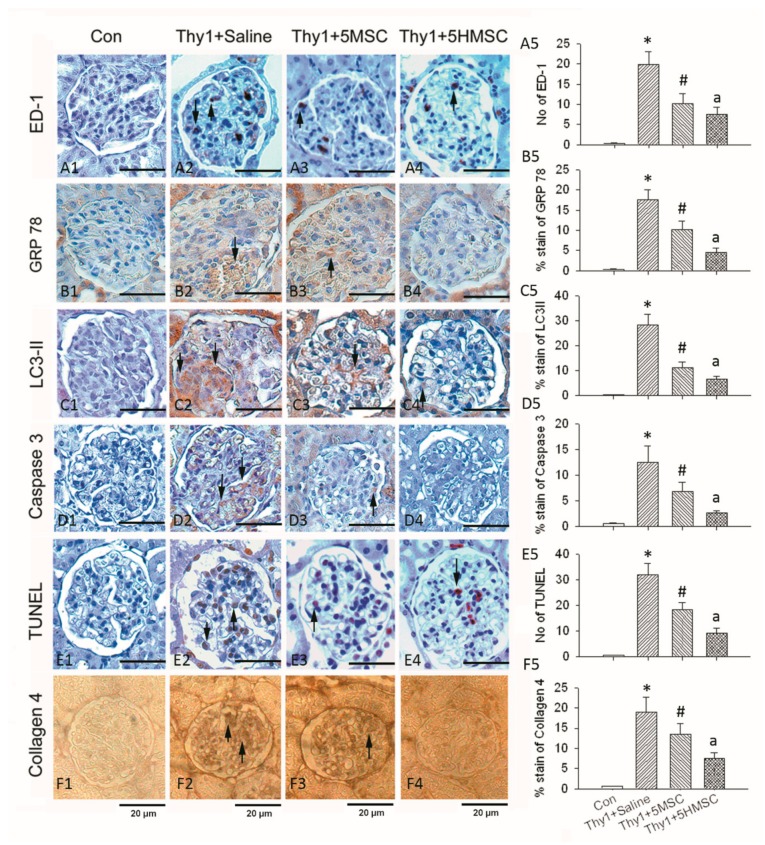
Immunohistochemical stains for ED1, GRP78, LC3-II, Caspase1, TUNEL, and Collagen IV in the study groups. Detection of ED1+ cell in the study groups are demonstrated in (**A1–4**). Semiquantitative assessment revealed infusion of MSCs and showed a reduction in numbers of macrophage/monocyte infiltration (ED1+ cell). HMSCs possessed a better reduction effect (**A5**). Representative pictures of sections for stress index protein acculumation (**B**1–4), autophagy by LC3-II (**C1–4**), apoptotic cells by caspase 1(**D1–4**) and TUNEL (**E1–4**) and collagen deposition (**F1–4**) in glomeruli. The semiquantitative assessment of these sections revealed anti-Thy1.1 administration increased stress index protein (GRP78) accumulation, autophagy index protein (LC3II) detection, apoptotic (TUNEL, caspase 1) cells, and collagen IV accumulation. MSC infusion ameliorated the increase in GRP78, LC3 II, caspase 1, TUNEL, and collagen IV in kidneys after anti-Thy1.1 infusion. HMSC infusion had greater therapeutic reduction effects. Each graph is amplified at 400×. The scale bar (20 μm) is indicated in the Figure. * *p* < 0.05 vs. Con group. # *p* < 0.05 vs. Thy1 group. a *p* < 0.05 vs. Thy1 + 5MSC group.

The representative pictures of immunohistochemical stained sections and semi-quantitative analyses of ED-1, GRP78, LC3-II, caspase 3, TUNEL and collagen IV in the study groups are presented in Figure 5. With the semi-quantitative analysis of the histochemical stained kidney sections for ED-1, anti-Thy1.1 administration markedly increased the numbers of ED-1positive cells in the glomeruli. Infusion of MSCs reduced the number of ED-1 positive cells that infiltrated in the kidneys, and infusion of HMSCs had a better reduction of the ED1 positive cell number (Figure 5A1–5) than MSCs. Similar results were found for GRP78, LC3-II, caspase 3, TUNEL, and collagen IV. These sections show that anti-Thy1.1 administration increased stress index protein (GRP78) accumulation (Figure 5B1–5), autophagy index protein (LC3-II) detection (Figure 5C1–5), caspase 3 positive cells (Figure 5D1–5), apoptotic (TUNEL+) cells (Figure 5E1–5), and collagen IV accumulation (Figure 5F1–5). MSC infusion ameliorated the increase in GRP78, LC3-II, caspase 3, TUNEL, and collagen IV in kidneys after anti-Thy1.1 infusion. HMSC infusion had a greater effect on ameliorating inflammatory cell infiltration, stress protein accumulation, apoptotic cells and autophagy in glomeuli vs. MSC treatment.

### 3.6. HMSCs Promote Nuclear Nrf2 Expression, Reduce NF-kB Expression, Rescue ROS Enzymatic Scavengers and Elevate Anti-Oxidative Response Element Proteins

**Figure 6:**
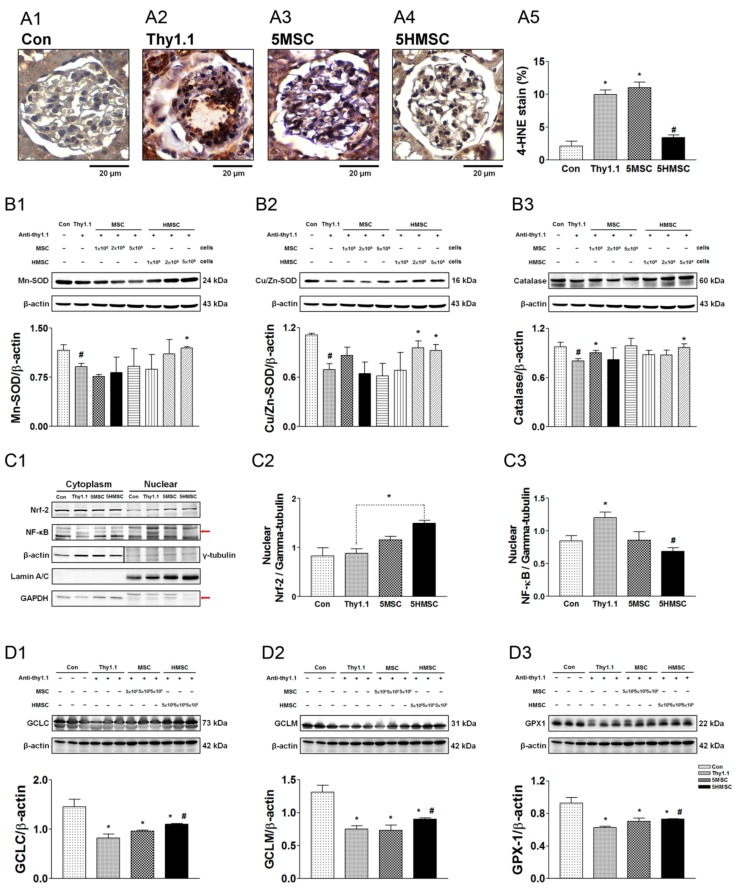
The index of ROS, ROS enzymatic scavenger expression, master anti-oxidative injury regulator Nrf2 expression, and anti-oxidative response protein expressions among the experimental groups. The accumulation of ROS by 4 HNE stain among the groups are demonstrated from the amplified graph at 400× (scale bar = 20 μm, **A1–4**). The semi-quantitative analyses of these sections revealed that anti-Thy1.1 infusion greatly increased ROS, and HMSCs ameliorated the ROS accumulation (**A5**). ROS enzymatic scavengers including MnSOD, Cu/ZnSOD and catalase were significantly reduced by antiThy-1 infusion. These enzymes were rescued by HMSCs (**B1–3**). The nuclear Nrf2 expression is not triggered by anti-Thy 1.1 infusion and is enhanced in the HMSC treated group. Significantly elevated nuclear NFkB expression is noted in anti-Thy1.1 group, which is efficiently suppressed in HMSCs treated group (**C1–3**). Downstream antioxidative response element protein expression including GCLC, GCLM, GPX are suppressed by anti-Thy 1.1 infusion. These proteins are rescued by HMSC transplantation (**D1–3**). * *p* < 0.05 vs. Con group. # *p* < 0.05 vs. Thy1.1 group.

Figure 6 demonstrates the reactive oxygen species (ROS) injury index and the intrinsic anti-oxidative mechanisms expression among the experimental groups. The accumulation of ROS by 4 HNE histochemical stain among the groups are demonstrated in Figure 6A1–4, the semi-quantitative analyses of these sections revealed that anti-Thy1.1 infusion greatly increased ROS. In the treatment groups, only a higher HMSC cell number ameliorated the ROS accumulation (Figure 6A5). ROS enzymatic scavenger expressions including MnSOD, Cu/ZnSOD, and catalase were significantly reduced by antiThy-1.1 infusion. These enzymes were rescued by HMSC transplantation, but not by MSCs (Figure 6B1–3). As for Nrf2 signaling, the master regulator of ROS injury, the results showed that nuclear Nrf2 expression was not changed, while NF-kB expression was elevated by anti-Thy1.1 infusion. Infusion of HMSCs significantly increased nuclear Nrf2 and reduced NF-kB expressions in anti-Thy1.1 treated rat kidneys. Infusion of MSCs had a lesser effect on nuclear Nrf2 elevation and NF-kB expression. However, the effect did not reach statistical significance (Figure 6C1–3). Semi-quantitative detection of anti-oxidative response element (ARE) protein expression (GCLC, GCLM and GPX) by Western blotting in the kidneys are shown (Figure 6D1–3). These proteins had a consistent expression trend in the experimental groups. Briefly, the anti-Thy1.1 antibody infusion reduced ARE protein expressions, and hypoxic preconditioning MSCs rescued these protein expressions. Administration of MSCs did not significantly increase protein expression, though it tended to increase their levels. Figure 6E demonstrated a summary diagram of this study.

## 4. Discussion

Hypoxic preconditioning significantly upregulated HIF-1α/VEGF expression in the MSCs, however, it downregulated several growth factors in the hypoxic conditioned medium implicating an enhanced HIF-1α/VEGF signaling. As far as we know, there is no in vivo data to confirm that HMSCs transplantation can upregulate HIF-1α and VEGF in the rat GN model. However, HMSCs transplantation increased expression of pro-survival and pro-angiogenic factors including HIF-1 and VEGF in the rat model of myocardial infarction [23] or ischemic/reperfusion kidney [24]. Our present data displayed that intrarenal arterial administration of either MSCs or HMSCs ameliorates the severity of glomerulosclerosis and levels of proteinuria in the anti-Thy-1.1 induced rat GN model. HMSCs showed a better therapeutic effect than MSCs on the amelioration of glomerulosclerosis, inflammatory cell infiltration, ER stress, apoptosis, and autophagy. In addition, hypoxic preconditioning enabled MSCs to activate nuclear Nrf2 expression and rescued ROS scavengers in kidneys after Thy-1.1 lesion. Our results indicate that hypoxic preconditioning further enhances the therapeutic effects of MSCs through multiple mechanisms including increasing intra-nuclear Nrf2 expression in the target organ.

Several studies have demonstrated that stem cells derived from various origins ameliorate kidney injury in GN animal models [2,25,26,27,28,29]. MSCs have the ability to cause anti-inflammation, anti-fibrosis, and inhibition of cell death and this is the basis for cell therapies [2,25,26,29,30]. In this study, both MSCs and HMSCs showed anti-inflammatory effects by decreasing macrophage/monocyte infiltration in glomeruli of treated kidneys and by inhibiting NF-κB translocation into nucleus. A significant further decrease in numbers was found in the HMSC treated group, indicating that hypoxic preconditioning is an effective strategy to promote the anti-inflammatory effect. The results of Masson stains confirm the therapeutic effect of MSCs on anti-fibrosis, and this effect was further enhanced by hypoxic preconditioning. We also demonstrated that intra-glomerular cell apoptosis and autophagy were decreased by MSC infusion, and a further reduction was noted in the HMSC treat group. The therapeutic anti-inflammation, anti-fibrosis, anti-apoptosis, and anti-autophagy mechanisms of MSCs are compatible to previous studies [31,32]. Hypoxic preconditioning showed a consistent enhancement of these therapeutic mechanisms seen in MSCs.

Another important finding in this study is that hypoxic preconditioning enabled MSCs to increase nuclear Nrf2 and decrease NF-κB expression. Oxidative stress generated by the immune reaction is believed to be one of the crucial mechanisms that cause injuries to glomeruli in the GN. The Keap1–Nrf2 pathway signaling and the anti-oxidant responsive elements play a central role in protection against oxidative stresses. In this study, anti-Thy1.1 lesion was found to suppress the antioxidant activity by decreasing ROS scavenger expression and elevating nuclear NF-κB, which contributed to inflammatory cytokine cascades. The nuclear Nrf2 expression remained unchanged after anti-Thy1.1 lesion, indicating that the master intrinsic anti-oxidative regulator, Nrf2/Keap1 pathway, was not triggered. Nrf2 expression levels increased with MSCs transplantation to GN rats. However, it failed to reach a significant difference. At the same time, ROS enzymatic scavengers and other ARE proteins were not rescued. With HMSC transplantation, nuclear Nrf2 expression increased and ROS scavengers and ARE proteins were rescued in diseased kidneys. From these results it seems that conventional MSC transplantation may not trigger enough Nrf2 pathway signaling activity to enhance ARE protein expressions. Therefore, hypoxic preconditioning enabled MSCs to activate the Nrf2 pathway signaling and to rescue the ROS scavengers in kidneys which were suppressed by the anti-Thy1.1 infusion. Our findings provide evidence supporting the viewpoint of Ezquer et al.’s study [33], in which MSCs were believed to possess the main enzymatic mechanisms to detoxify the reactive oxygen species and to prevent oxidative damage in rat nephritis based on some in vitro cellular studies [34,35].

The major challenges that underlie the application of stem cell therapy to GN patients are safety concerns and efficacy issues. Enhancing the anti-oxidative effect of stem cells is one promising strategy to promote their efficacy for inflammatory or oxidative stress related disease such as GN. Nrf2 is a crucial regulator of the antioxidant defense system and governs the expression of genes associated with redox homeostasis. The beneficial effects of targeting the Nrf2 pathway for nephritis have been demonstrated in animal studies through tranduction of the OR1 gene to enhance antioxidation [36] or via Keap-1 gene knockout to activate the Nrf2 system [37]. Bardoxolone, an Nrf2 activator, has been shown to improve renal functions in type 2 diabetic patients with chronic kidney disease in a human phase 2 trial [38], though it had cardiovascular safety issues [39]. The current study shows that HMSC transplantation is an effective measure to enhance Nrf2 pathway signaling and therapeutic effects in damaged kidneys. Hypoxic preconditioning, i.e., stem cell cultured in an ischemic condition which mimics the bone marrow niche environment, is a common way to preserve stemness, enhance homing, and increase efficacy of stem cell therapy. In cellular studies, hypoxic preconditioning has enhanced stemness and expanded cell numbers [40]. Hypoxic mimetic preconditioning enhances MSC migration and prolongs kidney retention through promoting CXCR4 expression [24]. The results of this study link hypoxic preconditioning to anti-oxidative injury. We demonstrated that HMSCs promote Nrf2 signaling and resultant ARE protein elevation. The cytokines involved in the Nrf2 pathway signaling activation promoted by HMSCs need to be further investigated.

MSC homing to injured tissue is the first step and crucial because the therapeutic application of stem cell therapy is predicated on the transplanted cells migrating and participating in tissue repair. Enhancing the homing capabilities and the self-defense potential of stem cells can promote their therapeutic efficacy. As shown in Figure 1C, the MSCs indeed transplanted to the damaged glomeruli implicating its efficient homing to the damaged site. Hypoxic preconditioning enhanced stem cells with several possible defense mechanisms including antioxidant, ant-apoptosis, anti-ER stress, and anti-inflammatory potential and the homing HMSC may protect itself and the adjacent cells against oxidative stress through the possible autocrine and/or paracrine effect to release growth factors and other protective mediators. This hypothesis requires further experiments to confirm. In future, we will perform the in vitro and in vivo experiments to determine the released protective molecules from the homing HMSCs and to explore the exact mechanism for attenuating glomerular injury in the GN model. In the present study, intrarenal arterial administrations of MSCs or HMSCs lead to positive CD44 staining existence in the glomeruli. The advantage of intrarenal arterial administration can demonstrate the direct delivery and location of stem cells to the kidney and prevent the risk of stem cells trapping in the lung or other non-target tissue/organ by systemically intravenous administration. The average stem cell numbers trafficked in glomeruli increased with hypoxic preconditioning and higher infused cell numbers. In previous studies, hypoxic precondition has been associated with increased CXCR4, CX3CR1 expression in a cellular study [17]. CXCR4 and CX3CR1 respond to SDF-1α, activate the Akt signal pathway [41] and elevate matrix metalloproteinases [42] contributing to transmigration. Our findings confirm that hypoxic preconditioning is an effective strategy to enhance the homing effect of MSCs in the rat GN model.

The mesangial cell proliferation peaks at about 1 week, and the nephritis spontaneously repairs after 2–3 weeks in the anti-Thy1.1 nephritic model. To prevent the possible spontaneous repair effect, we demonstrated the clinically available HMSCs transplantation on reducing glomerular matrix accumulation, attenuating proteinuria, and ameliorating glomerular sclerosis in rats with anti-Thy1 disease at the early stage within 1 week (sacrificed on day 5). These results suggest that renal arterial administration of HMSCs in vivo may have promise as an anti-inflammatory, anti-proliferative, and anti-fibrotic strategy in the treatment of acute phases or relapses of mesangial proliferative glomerulonephritis. There are some limitations to the current study. First, we only investigated the Nrf2 pathway and ARE expressions in the regulation of MSCs antioxidant status. Functions of other oxidative stress-related pathways, such as PI3K/Akt and FoxO/TXNIP need further elucidation. Secondly, mechanisms that influence the enhanced repairing efficacy of MSCs after transplantation were not fully elucidated. Further studies focusing on the cytokines involved in the anti-oxidant enhancement are needed. Third, we used an acute GN model in this study. Whether these results can be applied to chronic GN, mandates further investigations.

## 5. Conclusions

**Figure 7:**
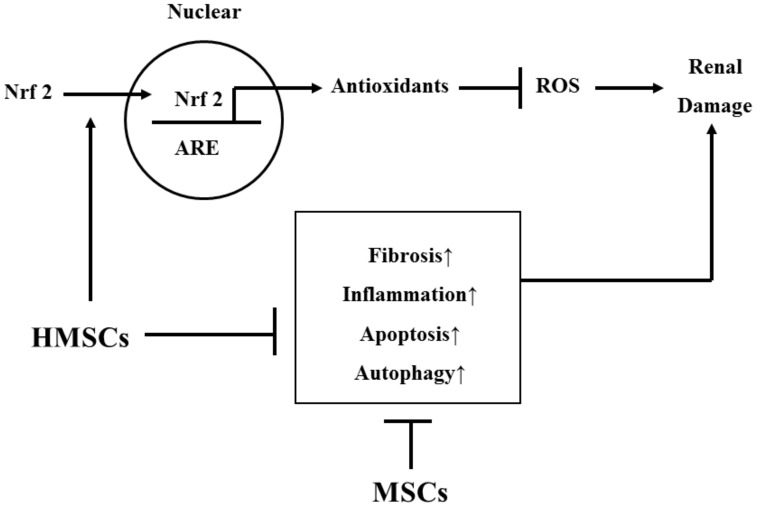
The summary diagram is demonstrated. HMSCs prevent renal damage by suppression of pathological signals (including fibrosis, inflammation, apoptosis, and autophagy) and increase antioxidant status against oxidative stress. HMSCs reduce renal damage majorly through suppression of pathological signals.

Our experimental data can be summarized as Figure 7. As well as enhancing the anti-inflammatory, anti-ER stress, anti-fibrosis, anti-apoptosis, and anti-autophagy properties, anti-oxidative mechanisms also play a role in the therapeutic effect of hypoxic preconditioned MSCs on glomerulonephritis. Hypoxic preconditioning is one effective strategy to activate further intrinsic anti-oxidative defense systems by promoting the HIF-1α/VEGF signaling, Nrf2 pathway, rescue antioxidant enzymes, and increase anti-oxidative responsive element proteins.

## Abbreviations

ED-1	macrophage/monocyte
ER stress	endoplasmic reticulum stress
ESRD	end stage renal disease
GN	glomerulonephritis
HIF-1α	hypoxia induced factor-1α
HMSCs	hypoxic-preconditioned mesenchymal stem cells
MSCs	mesenchymal stem cells
NF-kB	nuclear factor kappa B
Nrf2	nuclear factor (erythroid-derived 2)
ROS	reactive oxygen species
VEGF	vascular endothelial growth factor
TUNEL	terminal deoxynucleotidyl transferase–mediated digoxigenin-deoxyuridine nick-end labeling
